# Quality of social and emotional wellbeing services for families of young Indigenous children attending primary care centers; a cross sectional analysis

**DOI:** 10.1186/s12913-018-2883-6

**Published:** 2018-02-09

**Authors:** Karen M. Edmond, Kimberley McAuley, Daniel McAullay, Veronica Matthews, Natalie Strobel, Rhonda Marriott, Ross Bailie

**Affiliations:** 1 School of Medicine, Division Paediatrics, The University of Western Australia, 35 Stirling Highway, Crawley, Western Australia 6009 Australia; 2 Edith Cowen University, Perth, Western Australia Australia; 3 University Centre for Rural Health, University of Sydney, 61 Uralba Street, Lismore, New South Wales 2480 Australia; 4 School of Psychology and Exercise Science, Murdoch University, 90 South Street, Murdoch, Western Australia 6150 Australia

**Keywords:** Health services, Indigenous, Child health services, Quality improvement, Child welfare

## Abstract

**Background:**

The quality of social and emotional wellbeing services for Indigenous families of young children is not known, in many settings especially services provided by primary care centers.

**Methods:**

Our primary objective was to assess delivery of social and emotional wellbeing services to the families of young (3–11 months) and older (12–59 months) Indigenous children attending primary care centers. Our secondary objective was to assess if delivery differed by geographic location. Two thousand four hundred sixty-six client files from 109 primary care centers across Australia from 2012 to 2014 were analysed using logistic regression and generalised estimating equations.

**Results:**

The proportion of families receiving social and emotional wellbeing services ranged from 10.6% (102) (food security) to 74.7% (1216) (assessment of parent child interaction). Seventy one percent (71%, 126) of families received follow up care. Families of children aged 3–11 months (39.5%, 225) were more likely to receive social and emotional wellbeing services (advice about domestic environment, social support, housing condition, child stimulation) than families of children aged 12–59 months (30.0%, 487) (adjusted odds ratio [aOR] 1.68 95% CI 1.33 to 2.13). Remote area families (32.6%, 622) received similar services to rural (29.4%, 68) and urban families (44.0%, 22) (aOR 0.64 95% CI 0.29, 1.44).

**Conclusions:**

The families of young Indigenous children appear to receive priority for social and emotional wellbeing care in Australian primary care centers, however many Indigenous families are not receiving services. Improvement in resourcing and support of social and emotional wellbeing services in primary care centers is needed.

**Electronic supplementary material:**

The online version of this article (10.1186/s12913-018-2883-6) contains supplementary material, which is available to authorized users.

## Background

Primary care providers work at the first level of the health system in clinics and health centers and include nurses, doctors and community health workers. Evidence is emerging about the potential for primary care providers to improve social and emotional wellbeing and long term neurodevelopmental outcomes in disadvantaged children [1–4]. ‘Child health checks’ are a core component of primary care for Aboriginal and Torres Strait Islander (hereafter Indigenous) children across Australia [5–7]. The governments in all Australian states and territories advise primary care providers to administer at least one ‘child health check’ to each Indigenous child every 12 months [5, 6, 8]. The checks are standardised, based on best practice national guidelines, and include physical measurements such as weight and height, assessment of oral health, assessment of developmental milestones and discussion about social and emotional needs including: parent child interaction, physical and mental stimulation of the child, child behaviour, optimal domestic and social environment, housing and food security, and social and family support services [8].

However, it is not clear how well social and emotional wellbeing services are being implemented in busy primary care settings, and there is little information about the implementation of social and emotional services for the families of the youngest infants who require the most support. Variation between urban, rural and remote locations is also unclear.

The Audit and Best Practice for Chronic Disease (ABCD) program is a continuous quality improvement (CQI) program for the prevention and management of chronic disease in Indigenous people in Australia [9, 10]. The program broadened its scope in 2007 to include maternal and child health, mental health and health promotion. By the end of 2014 ABCD and its service support organisation (One21seventy [11]) had supported over 270 primary care centers across Australia. There are now data available from standardised audits of almost 15,000 clinical files of Indigenous children who attended primary care centers across Australia from 2007 to 2014.

The primary objective of this study was to use ABCD data from 2012 to 2014 to assess the delivery of social and emotional wellbeing services in Australian primary care centers to the families of young (3–11 months) and older (12–59 months) Indigenous children. The secondary objective was to assess if delivery differed by geographic location.

## Methods

### Study setting and design

This was a cross sectional study of client files from the 109 remote, rural and urban Australian primary care centers that participated in ABCD from 2012 to 2014. Key characteristics are presented in Table 1.

**Table 1:** Key characteristics of client files by geographic location, age and CQI participation in Indigenous children aged 3–59 months

		Geographic location			Age (months)			CQI participation (number of audits completed)		
	Total	Remote	Rural	Urban	3–11	12–23	24–59	1	2	> = 3
Total	2466	2010 (81.51%)	371 (15.0%)	85 (3.5%)	609 (24.7%)	532 (21.6%)	1325 (53.7%)	410 (16.6%)	569 (23.1%)	1487 (60.3%)
Health service characteristics										
Governance										
Aboriginal community controlled	573 (23.2%)	319 (15.9%)	224 (60.4%)	30 (35.3%)	133 (21.8%)	118 (22.2%)	322 (24.3%)	77 (18.8%)	182 (32.0%)	314 (21.1%)
Government	1893 (76.8%)	1691 (84.1%)	147 (39.6%)	55 (64.7%)	476 (78.2%)	414 (77.8%)	1003 (75.7%)	333 (81.2%)	387 (68.0%)	1173 (78.9%)
Health service provider who first saw the child										
Indigenous health worker	338 (13.7%)	220 (10.9%)	94 (25.3%)	24 (28.2%)	69 (11.3%)	67 (12.6%)	202 (15.2%)	21 (5.1%)	88 (15.5%)	229 (15.4%)
Nurse	1723 (69.9%)	1505 (74.9%)	176 (47.4%)	42 (49.4%)	460 (75.5%)	381 (71.6%)	882 (66.6%)	286 (69.8%)	430 (75.6%)	1007 (67.7%)
General practitioner	271 (11.0%)	163 (8.1%)	90 (24.3%)	18 (21.2%)	62 (10.2%)	60 (11.3%)	149 (11.2%)	81 (19.8%)	27 (4.7%)	163 (11.0%)
Other	117 (4.7%)	106 (5.3%)	10 (2.7%)	1 (1.2%)	16 (2.6%)	20 (3.8%)	81 (6.1%)	21 (5.1%)	24 (4.2%)	72 (4.8%)
Missing	17 (0.7%)	16 (0.8%)	1 (0.3%)	0 (0.0%)	2 (0.3%)	4 (0.8%)	11 (0.8%)	1 (0.2%)	0 (0.0%)	16 (1.1%)
Year of data collection										
2012	488 (19.8%)	319 (15.9%)	148 (39.9%)	21 (24.7%)	127 (20.9%)	107 (20.1%)	254 (19.2%)	196 (47.8%)	84 (14.8%)	208 (14.0%)
2013	1334 (54.1%)	1163 (57.9%)	171 (46.1%)	0 (0.0%)	313 (51.4%)	276 (51.9%)	745 (56.2%)	155 (37.8%)	274 (48.2%)	905 (60.9%)
2014	644 (26.1%)	528 (26.3%)	52 (14.0%)	64 (75.3%)	169 (27.8%)	149 (28.0%)	326 (24.6%)	59 (14.4%)	211 (37.1%)	374 (25.2%)
Population size										
< 500	848 (34.4%)	831 (41.3%)	17 (4.6%)	0 (0.0%)	137 (22.5%)	196 (36.8%)	515 (38.9%)	101 (24.6%)	175 (30.8%)	572 (38.5%)
500–999	499 (20.2%)	448 (22.3%)	41 (11.1%)	10 (11.8%)	114 (18.7%)	101 (19.0%)	284 (21.4%)	104 (25.4%)	92 (16.2%)	303 (20.4%)
> =1000	1119 (45.4%)	731 (36.4%)	313 (84.4%)	75 (88.2%)	358 (58.8%)	235 (44.2%)	526 (39.7%)	205 (50.0%)	302 (53.1%)	612 (41.2%)
Child characteristics										
Sex of child										
Male	1249 (50.6%)	1017 (50.6%)	189 (50.9%)	43 (50.6%)	310 (50.9%)	272 (51.1%)	667 (50.3%)	202 (49.3%)	283 (49.7%)	764 (51.4%)
Female	1217 (49.4%)	993 (49.4%)	182 (49.1%)	42 (49.4%)	299 (49.1%)	260 (48.9%)	658 (49.7%)	208 (50.7%)	286 (50.3%)	723 (48.6%)
Type of child health check completed in the last 12 months										
Medical benefits schedule (MBS) 715	999 (40.5%)	847 (42.1%)	122 (32.9%)	30 (35.3%)	246 (40.4%)	229 (43.0%)	524 (39.5%)	115 (28.0%)	212 (37.3%)	672 (45.2%)
Other child health check	648 (26.3%)	507 (25.2%)	124 (33.4%)	17 (20.0%)	181 (29.7%)	147 (27.6%)	320 (24.2%)	144 (35.1%)	88 (15.5%)	416 (28.0%)
Not known / not recorded	819 (33.2%)	656 (32.6%)	125 (33.7%)	38 (44.7%)	182 (29.9%)	156 (29.3%)	481 (36.3%)	151 (36.8%)	269 (47.3%)	399 (26.8%)
Reason for last clinic attendance										
Acute care	1200 (48.7%)	990 (49.3%)	171 (46.1%)	39 (45.9%)	265 (43.5%)	271 (50.9%)	664 (50.1%)	161 (39.3%)	284 (49.9%)	755 (50.8%)
Vaccination	366 (14.8%)	268 (13.3%)	78 (21.0%)	20 (23.5%)	122 (20.0%)	87 (16.4%)	157 (11.8%)	64 (15.6%)	89 (15.6%)	213 (14.3%)
Child health check	577 (23.4%)	467 (23.2%)	92 (24.8%)	18 (21.2%)	155 (25.5%)	112 (21.1%)	310 (23.4%)	105 (25.6%)	117 (20.6%)	355 (23.9%)
Other	323 (13.1%)	285 (14.2%)	30 (8.1%)	8 (9.4%)	67 (11.0%)	62 (11.7%)	194 (14.6%)	80 (19.5%)	79 (13.9%)	164 (11.0%)

*CQI* Continuous quality improvement

### Data collection

The ABCD program included annual audits of client files in all participating primary care centers [9, 10]. The audits were implemented by primary care center staff who had received standardised training with ABCD educators including assessment of interrater reliability [9, 10]. The training program was based on national best practice clinical guidelines and involved face to face training sessions using a standardised manual and data collection tool which are available online [10, 12]. Client files were eligible if the following criteria were met: i) child aged between 3 months and 14 years at the audit date; ii) child resident in the community for at least 6 months (or half of the time since birth if aged under 6 months); and iii) child had no major health anomaly such as heart defects or inherited disorders.

A random sample of at least 30 individual patient records were selected for audit from each health center. The auditors read each client file (electronic and paper) and recorded information in a standardised pre-coded data collection tool. Child characteristics included: date of birth, age, sex, Indigenous status, attendance at the primary care center in the previous 12 months, reason for the last attendance (acute care, health check, vaccination, other) and receipt of any child health checks in the last 12 months (Australian Commonwealth funded [Medicare 715] or other child health check). Health center characteristics included governance (Aboriginal community controlled health service or government operated), location (urban, rural or remote), and number of CQI audits the primary care center had completed.

The ABCD audit tool also included eleven pre-coded items about social and emotional wellbeing services (Table 2). The auditors scored ‘yes’ in the audit tool if there had been any documentation in the client file in the last 12 months of: (i) assessment of parent child interaction (e.g. bonding, attachment, responsiveness); (ii) advice about: childhood domestic or social environment (e.g. violence, substance use, gambling); social or family support (e.g. other family members involved in care of child, attendance at parent groups, social worker involvement); finances (e.g. regular employment, social service payments, food cards); housing condition (e.g. overcrowding, water and sanitation); food security (e.g. food consumed in the last 24 h and in the last week); physical and mental stimulation (e.g. play, reading, attendance at play groups); child behaviour (e.g. sleep, crying, and temper tantrums); (iii) evidence of concern about: domestic or social environment; social or family support; housing condition; and food security; (iv) follow up or referral regarding concern about domestic or social environment; social or family support; housing condition and food security. Items were recorded as ‘not applicable’ if they were not specified in the national best practice guidelines for children of that age [5–7]. The items that were specified in the national best practice guidelines for all families of children aged between 3 and 59 months were advice about: physical and mental stimulation for children; domestic or social environment; social or family support and advice about housing condition.

**Table 2:** Social and emotional wellbeing care for families of Indigenous children aged 3–59 months by geographic location, age and CQI participation

				Geographic location			Age (months)			CQI participation (number of audits completed)		
	Number of eligible primary care centers n (%)	Number of client records assessed n (%)^a^	Total receiving care n (%)	Remote n (%)	Rural n (%)	Urban n (%)	3–11 n (%)	12–23 n (%)	24–59 n (%)	1 n (%)	2 n (%)	> = 3 n (%)
Total	109	2466	2466	2010	371	85	609	532	1325	410	569	1487
Assessment												
Assessment of parent-child interaction	109 (100%)	1628 (66.0%)	1216 (74.7%)	986 (78.9%)	198 (65.4%)	32 (42.1%)	516 (84.9%)	389 (73.3%)	311 (63.6%)	155 (59.6%)	231 (73.3%)	830 (78.8%)
Anticipatory guidance												
Advice about domestic/social environment	109 (100%)	2466 (100%)	1544 (62.6%)	1276 (63.5%)	221 (59.6%)	47 (55.3%)	427 (70.1%)	368 (69.2%)	749 (56.5%)	201 (49.0%)	293 (51.5%)	1050 (70.6%)
Advice about social/family support	109 (100%)	2466 (100%)	1410 (60.4%)	1162 (60.8%)	205 (60.3%)	43 (53.1%)	414 (68.0%)	367 (69.0%)	629 (52.8%)	182 (47.9%)	281 (49.6%)	947 (68.3%)
Advice about financial situation	109 (100%)	1373 (55.7%)	236 (17.2%)	210 (16.8%)	5 (5.6%)	21 (70.0%)	79 (23.5%)	62 (21.8%)	95 (12.6%)	61 (23.8%)	35 (7.7%)	140 (21.1%)
Advice about housing condition	109 (100%)	2466 (100%)	1140 (46.2%)	939 (46.7%)	169 (45.6%)	32 (37.7%)	327 (53.7%)	255 (47.9%)	558 (42.1%)	167 (40.7%)	245 (43.1%)	728 (49.0%)
Advice about food security	109 (100%)	963 (39.1%)	102 (10.6%)	73 (9.7%)	23 (12.3%)	6 (25.0%)	39 (15.9%)	29 (13.9%)	34 (6.7%)	10 (8.6%)	0 (0.0%)	92 (11.1%)
Advice about physical and mental stimulation of child	109 (100%)	2322 (94.2%)	1279 (55.1%)	1137 (56.7%)	102 (38.9%)	40 (74.1%)	366 (64.2%)	312 (52.0%)	601 (48.1%)	135 (36.3%)	222 (46.7%)	922 (62.5%)
Advice about child behaviour (e.g temper tantrums, sleep disturbance)	109 (100%)	963 (39.1%)	703 (73.0%)	584 (77.7%)	102 (54.6%)	17 (70.8%)	211 (85.8%)	170 (81.3%)	322 (63.4%)	65 (56.0%)	20 (100.0%)	618 (74.7%)
Follow up of problems and concerns												
Clinic follow up and/or referral for problems with domestic environment	109 (100%)	111 (4.50%)	82 (73.9%)	23 (26.7%)	3 (16.7%)	3 (42.9%)	8 (32.0%)	7 (23.3%)	14 (25.0%)	16 (84.2%)	9 (52.9%)	57 (76.0%)
Clinic follow up and/or referral for family and financial support	109 (100%)	63 (2.55%)	46 (73.0%)	13 (28.9%)	2 (16.7%)	2 (33.3%)	4 (30.8%)	7 (33.3%)	6 (20.7%)	15 (68.2%)	5 (71.4%)	26 (76.5%)
Clinic follow up and/or referral for housing condition or food security	109 (100%)	80 (3.24%)	55 (68.8%)	22 (31.9%)	2 (25.0%)	1 (33.3%)	6 (33.3%)	8 (36.4%)	11 (27.5%)	14 (60.9%)	6 (50.0%)	35 (77.8%)
Composite measure of quality of care^b^	109 (100%)	2189 (88.8%)	712 (32.5%)	622 (32.6%)	68 (29.4%)	22 (44.0%)	225 (39.5%)	174 (34.6%)	313 (28.0%)	81 (23.7%)	116 (24.5%)	515 (37.5%)

*CQI* Continuous quality improvement

^a^Proportions are less than 100% if the service is not included in the best practice guidelines for children of that age

^b^Families received advice about domestic environment, social support, housing condition and child stimulation

### Definitions

We defined a composite measure of social and emotional wellbeing care for the four items required in the national guidelines for all children aged 3–59 months i.e. advice provided at least once in the last 12 months about: domestic environment, social support, housing condition and child stimulation. The composite measure was scored as ‘yes’ if all four areas were documented in the client file. We explicitly planned to only include items in the composite score that were required for all families of children under 5 years. The auditors read each client file (electronic and paper) and recorded information in a standardised pre-coded data collection tool for all (100%) of health centres which included the item in their best practice guidelines.

We divided geographic location into three categories based on the Accessibility/ Remoteness Index of Australia (ARIA) [13]. ARIA data are split into five categories from least remote (1) (major cities) to most remote (5) (remote area communities). We defined ‘urban’ as ARIA category one, ‘rural’ as ARIA categories two to four and ‘remote’ as ARIA category five. The urban category was very small (Table 1) so we combined rural and urban into a ‘non-remote’ category for all statistical analyses.

‘Any CQI participation’ was defined as the completion of at least one ABCD audit. ‘High CQI participation’ was defined as completing three or more ABCD audits.

### Statistical analysis

We only included the last audit conducted by each health centre between 2012 and 2014, so each health centre was only represented once within the time period and each client record was included only once in the analysis. All other records were excluded.

The primary outcome measure was the proportion of families which had received the composite measure of social and emotional wellbeing care. We calculated that our dataset of almost 2500 client files would provide 90% power to detect at least a 10% difference in quality of care for families of children aged 3–11 months and families of children aged 12–59 months. We assumed a 5% significance level, a baseline level of quality of care of 50% in families children aged 3–11 months and that the ratio between families of children aged 3–11 months and 12–59 months would be approximately 1:4 [14].

The items assessed in this study are services provided for the whole client population of both well and unwell children. The denominator is the client population and the numerator is whether a problem is detected or not as a dichotomous variable. Crude and adjusted logistic generalised estimating equations (GEE) were used to examine the effect of age (3–11, 12--59 months) on the delivery of social and emotional wellbeing care and to account for loss of independence due to clustering. GEEs are a standard method widely recommended to account for loss of independence in regression models due to clustering [15–17]. Odds ratios (ORs) and 95% confidence intervals (95% CI) were calculated. Multilevel binomial models with an exchangeable correlation structure and robust standard errors were used with primary care center as the clustering variable. Multivariable regression models were constructed a priori to adjust for the effect of important explanatory variables: age, sex of child, geographic location, governance structure, CQI participation andk year of data collection. Similar methods were used to assess if effects differed by geographic location. Data analyses were conducted using STATA 13.1.

### Ethical approval

Ethical approval was obtained from all Human Research Ethics Committees (HRECs) in the states and territories involved: the Human Research Ethics Committee (HREC) of the Northern Territory Department of Health and Menzies School of Health Research (HREC-EC00153); Central Australian HREC (HREC-12-53); Queensland HREC Darling Downs Health Services District (HREC/11/QTDD/47); South Australian Indigenous Health Research Ethics Committee (04–10-319); Curtin University HREC (HR140/2008); Western Australian Country Health Services Research Ethics Committee (2011/27); Western Australian Aboriginal Health Ethics Committee (111–8/05); and University of Western Australia HREC (RA/4/1/5051).

## Results

There were 2466 client files of Indigenous children aged between 3 and 59 months from 109 primary care centers across five Australian states and territories (Northern Territory, Queensland, New South Wales, South Australia and Western Australia) from 2012 to 2014 (Table 1). The mean age of children was 27.3 months (standard deviation 16.8) and the median was 25 months (interquartile range 12 to 42). Eighty one percent (2010, 81.5%) of audits were performed in remote areas and only 3.5% (85) in urban areas. 60.3% (1487) of audits were conducted in clinics that had completed three or more audits (Table 1). There was little difference in the characteristics of health centers that reviewed young (3–11 months) and older (12–59 months) children (Table 1). There were also few differences between the characteristics of health centers in remote and non remote locations and those that participated in one or more ABCD audits (Table 1, Additional file 1: Tables S1-S4).

All 100% (109) of primary care centers included the eleven age specific social and emotional wellbeing items in their clinic protocols (Table 2). However, only 37 % (37.5%, 515) of families received the composite measure of quality of care (advice about domestic environment, social support, housing condition and child stimulation). The proportion of families who received specific services ranged from 10.6% (102) (advice about food security) to 74.7% (1216) (assessment of parent child interaction) (Table 2). Sixty two percent (62.5%, 922) of families received advice about child stimulation. Seventy five percent (74.7%, 618) received advice about child behaviour. Almost 76% (75.9%, 57) received clinic follow up or referral for concerns about domestic environment, 76% (76.5%, 26) received follow up for concerns about family support and financial situation and almost 78% (77.8%, 35) received follow up for concerns about housing condition and food security.

Age of the child was strongly associated with quality of social and emotional wellbeing care (Tables 2 and 3). Families of children aged 3–11 months (39.5%, 225) were more likely to receive the composite measure (advice about domestic environment, social support, housing condition, child stimulation) than families of children aged 12–59 months (30.0%, 487) (adjusted odds ratio [aOR] 1.68 95% CI 1.33 to 2.13). Families of children aged 3–11 months were also more likely to receive the composite measure than families of children aged 12–23 months (34.6%, 174) (aOR 1.39 95% CI 1.11, 1.78) and families of children aged 24–59 months (28.0%, 313) (aOR 1.89 95% CI 1.42, 2.51).

**Table 3:** Association between key characteristics and social and emotional wellbeing care for families of Indigenous children aged 3–59 months

	Number of client records n (%)	Number of client records with composite measure n (%)	OR (95% CI)	*P* value	aOR^a^ (95% CI)	*P* value
	2189	712 (32.5%)				
Health service characteristics						
Geographic location						
Remote	1908	622 (32.6%)	1.00		1.00	
Non remote	281	90 (32.0%)	0.69 (0.33,1.44)	0.321	0.64 (0.29,1.44)	0.286
CQI participation (number of audits completed)						
1	342	81 (23.7%)	1.00		1.00	
2	473	116 (24.5%)	1.31 (0.48,3.60)	0.597	0.86 (0.30,2.47)	0.785
> = 3	1374	515 (37.5%)	1.92 (0.78,4.71)	0.153	1.45 (0.61,3.46)	0.398
Governance						
Aboriginal community controlled health service	460	172 (37.4%)	1.35 (0.75,2.44)	0.323	1.40 (0.74,2.63)	0.298
Government health service	1729	540 (31.2%)	1.00		1.00	
Health service provider who first saw the child						
Indigenous health worker	265	94 (35.5%)	1.00 (0.79,1.26)	0.985	0.99 (0.78,1.24)	0.919
Nurse	1567	509 (32.5%)	1.00		1.00	
General practitioner	235	69 (29.4%)	0.76 (0.56,1.04)	0.086	0.76 (0.56,1.04)	0.083
Other	108	36 (33.3%)	0.97 (0.59,1.58)	0.888	1.05 (0.64,1.71)	0.850
Missing	14	4 (28.6%)				
Year of data collection						
2012	329	103 (31.3%)	1.00		1.00	
2013	1235	368 (29.8%)	0.87 (0.43,1.76)	0.693	0.79 (0.39,1.60)	0.506
2014	625	241 (38.6%)	1.35 (0.65,2.83)	0.421	1.38 (0.65,2.94)	0.396
Population size						
< 500	801	270 (33.7%)	1.00		1.00	
500–999	433	156 (36.0%)	1.17 (0.67,2.05)	0.575	1.18 (0.66,2.11)	0.579
> =1000	955	286 (29.9%)	1.02 (0.58,1.81)	0.935	0.96 (0.51,1.82)	0.911
Child characteristics						
Age of child						
3-11 m	570	225 (39.5%)	1.86 (1.41,2.46)	< 0.001	1.89 (1.42,2.51)	< 0.001
12-23 m	503	174 (34.6%)	1.38 (1.08,1.75)	0.009	1.39 (1.08,1.78)	0.009
24-59 m	1116	313 (28.0%)	1.00		1.00	
Sex of child						
Male	1109	376 (33.9%)	1.00		1.00	
Female	1080	336 (31.1%)	0.91 (0.76,1.08)	0.287	0.92 (0.77,1.09)	0.329
Type of child health check completed in the last 12 months						
Medical benefits schedule (MBS) 715	915	394 (43.1%)	1.00		1.00	
Other child health check	576	186 (32.3%)	0.77 (0.56,1.05)	0.1.00	0.74 (0.53,1.02)	0.067
Not known / not recorded	698	132 (18.9%)	0.41 (0.30,0.56)	< 0.001	0.42 (0.30,0.59)	< 0.001
Reason for last clinic attendance						
Acute care	1087	347 (31.9%)	0.79 (0.62,0.99)	0.043	0.81 (0.63,1.02)	0.078
Vaccination	300	74 (24.7%)	0.84 (0.63,1.12)	0.241	0.79 (0.58,1.07)	0.127
Child health check	507	183 (36.1%)	1.00		1.00	
Other	295	108 (36.6%)	0.85 (0.62,1.17)	0.324	0.92 (0.67,1.28)	0.622

*OR* Odds ratio, *aOR* adjusted odds ratio ^a^Adjusted for age, sex, year of data collection, geographic location, governance, CQI participation

Geographic location was not associated with quality of care (Tables 2 and 3). Families living in remote areas (32.6%, 622) had a similar composite measure to families living in rural (29.4%, 68) and urban areas (44.0%, 22) (aOR 0.64 95% CI 0.29, 1.44). Families attending centers with high CQI participation (completed three or more ABCD audits) had a similar composite measure (37.5%, 515) to families attending centers which had completed only one audit (23.7%, 81) (aOR 1.45 95% CI 0.61, 3.46) (Table 2). In clinics with high CQI participation, delivery ranged from 11.1% (92) (advice about nutrition and food security) to 78.8% (830) (assessment of parent child interaction) (Table 2). Sixty two percent (62.5%, 922) of families received advice about stimulation of their child. Seventy five percent (74.7%, 618) received advice about child behaviour. 71% (126) of families received follow up care.

Only 4.5% (111) of families were reported to have concerns about domestic environment, 2.6% (63) had concerns about family support and 3.2% (80) had concerns about housing and food security (Table 4). Two percent of families (56, 2.3%) had two or more concerns. Overall 92.8% (2288) of families were reported to have no concerns. Age of the child was not associated with reporting of concerns (Table 4). The proportion of families who had at least one concern was also similar in remote (7.1%, 143) and non remote areas (7.7%, 35) and in centers with high and low CQI participation (Table 4).

**Table 4:** Associations between key characteristics and abnormal findings in families of Indigenous children aged 3–59 months

		Problems with domestic environment			Problems with family and financial support			Problems with housing condition and food security		
	Number of client records	Number with problems n (%)	OR (95% CI)	aOR^a^ (95% CI)	Number with problems n (%)	OR (95% CI)	aOR (95% CI)	Number with problems n (%)	OR (95% CI)	aOR (95% CI)
Total	2466	111 (4.5%)			63 (2.6%)			80 (3.2%)		
Child characteristics										
Age of child										
3-11 m	609	25 (4.1%)	0.88 (0.52,1.49)	0.88 (0.53,1.47)	13 (2.1%)	0.77 (0.36,1.62)	0.77 (0.38,1.58)	18 (3.0%)	0.96 (0.52,1.78)	0.97 (0.53,1.78)
12-23 m	532	30 (5.6%)	1.28 (0.80,2.03)	1.29 (0.80,2.07)	21 (3.9%)	1.66 (0.95,2.88)	1.64 (0.95,2.83)	22 (4.1%)	1.34 (0.84,2.13)	1.34 (0.84,2.13)
24-59 m	1325	56 (4.2%)	1.00	1.00	29 (2.2%)	1.00	1.00	40 (3.0%)	1.00	1.00
Sex of child										
Male	1249	58 (4.6%)	1.00	1.00	28 (2.2%)	1.00	1.00	42 (3.4%)	1.00	1.00
Female	1217	53 (4.4%)	0.94 (0.67,1.32)	0.94 (0.67,1.32)	35 (2.9%)	1.28 (0.72,2.27)	1.27 (0.73,2.21)	38 (3.1%)	0.97 (0.52,1.80)	0.96 (0.52,1.79)
Type of child health check completed in the last 12 months										
MBS 715	999	55 (5.5%)	1.00	1.00	30 (3.0%)	1.00	1.00	38 (3.8%)	1.00	1.00
Other child health check	648	32 (4.9%)	0.94 (0.52,1.70)	1.00 (0.57,1.76)	18 (2.8%)	0.84 (0.42,1.69)	0.83 (0.41,1.66)	23 (3.5%)	0.79 (0.38,1.63)	0.81 (0.41,1.59)
Not known / not recorded	819	24 (2.9%)	0.56 (0.33,0.97)	0.58 (0.34,0.98)	15 (1.8%)	0.63 (0.32,1.24)	0.58 (0.32,1.06)	19 (2.3%)	0.72 (0.42,1.22)	0.70 (0.42,1.16)
Reason for last clinic attendance										
Acute care	1200	60 (5.0%)	1.26 (0.76,2.08)	1.26 (0.77,2.07)	29 (2.4%)	1.44 (0.73,2.81)	1.44 (0.76,2.74)	38 (3.2%)	1.50 (0.80,2.80)	1.49 (0.80,2.77)
Vaccination	366	12 (3.3%)	0.82 (0.42,1.63)	0.81 (0.42,1.56)	11 (3.0%)	1.63 (0.61,4.40)	1.54 (0.59,4.00)	11 (3.0%)	1.69 (0.71,4.03)	1.71 (0.72,4.03)
Child health check	577	21 (3.6%)	1.00	1.00	9 (1.6%)	1.00	1.00	11 (1.9%)	1.00	1.00
Other	323	18 (5.6%)	1.25 (0.62,2.51)	1.34 (0.66,2.73)	14 (4.3%)	2.01 (0.89,4.54)	2.01 (0.91,4.45)	20 (6.2%)	2.53 (1.13,5.70)	2.43 (1.08,5.48)
Health service characteristics										
Geographic location										
Remote	2010	86 (4.3%)	1.00	1.00	45 (2.2%)	1.00	1.00	69 (3.4%)	1.00	1.00
Non remote	456	25 (5.5%)	1.52 (0.74,3.10)	1.47 (0.51,4.18)	18 (3.9%)	2.52 (1.09,5.81)	1.92 (0.63,5.86)	11 (2.4%)	0.64 (0.26,1.56)	0.51 (0.20,1.32)
CQI participation (number of audits)										
1	410	19 (4.6%)	1.00	1.00	22 (5.4%)	1.00	1.00	23 (5.6%)	1.00	1.00
2	569	17 (3.0%)	0.63 (0.26,1.52)	0.81 (0.25,2.62)	7 (1.2%)	0.21 (0.09,0.50)	0.25 (0.09,0.71)	12 (2.1%)	0.38 (0.13,1.10)	0.27 (0.09,0.76)
> = 3	1487	75 (5.0%)	1.20 (0.54,2.65)	1.50 (0.52,4.34)	34 (2.3%)	0.43 (0.20,0.95)	0.53 (0.20,1.41)	45 (3.0%)	0.56 (0.25,1.26)	0.45 (0.21,0.98)
Governance										
Aboriginal community controlled health service	573	36 (6.3%)	1.65 (0.75,3.62)	1.43 (0.58,3.50)	21 (3.7%)	1.77 (0.70,4.49)	1.48 (0.54,4.11)	21 (3.7%)	1.29 (0.47,3.50)	1.38 (0.49,3.84)
Government health service	1893	75 (4.0%)	1.00	1.00	42 (2.2%)	1.00	1.00	59 (3.1%)	1.00	1.00
Health service provider who first saw the child										
Indigenous health worker	338	8 (2.4%)	0.49 (0.26,0.93)	0.45 (0.27,0.77)	7 (2.1%)	0.89 (0.42,1.90)	0.78 (0.41,1.50)	5 (1.5%)	0.54 (0.26,1.10)	0.54 (0.26,1.12)
Nurse	1723	83 (4.8%)	1.00	1.00	42 (2.4%)	1.00	1.00	62 (3.6%)	1.00	1.00
General practitioner	271	13 (4.8%)	0.78 (0.33,1.86)	0.73 (0.32,1.69)	10 (3.7%)	1.27 (0.48,3.35)	1.13 (0.43,3.00)	8 (3.0%)	0.56 (0.20,1.52)	0.56 (0.19,1.58)
Other	117	6 (5.1%)	1.11 (0.52,2.40)	1.09 (0.51,2.35)	4 (3.4%)	1.58 (0.64,3.88)	1.57 (0.65,3.76)	4 (3.4%)	0.93 (0.36,2.42)	0.92 (0.36,2.31)
Missing	17	1 (5.9%)			0 (0.0%)			1 (5.9%)		
Year of data collection										
2012	488	36 (7.4%)	1.00	1.00	23 (4.7%)	1.00	1.00	17 (3.5%)	1.00	1.00
2013	1334	44 (3.3%)	0.46 (0.21,0.99)	0.42 (0.19,0.93)	23 (1.7%)	0.33 (0.13,0.85)	0.45 (0.15,1.36)	37 (2.8%)	0.75 (0.24,2.30)	0.81 (0.24,2.81)
2014	644	31 (4.8%)	0.57 (0.25,1.28)	0.51 (0.22,1.18)	17 (2.6%)	0.49 (0.20,1.22)	0.57 (0.20,1.68)	26 (4.0%)	1.17 (0.37,3.67)	1.26 (0.37,4.23)
Population size										
< 500	848	26 (3.1%)	1.00	1.00	18 (2.1%)	1.00	1.00	33 (3.9%)	1.00	1.00
500–999	499	19 (3.8%)	1.21 (0.50,2.89)	1.17 (0.47,2.93)	6 (1.2%)	0.54(0.21,1.44)	0.42 (0.15,1.18)	6 (1.2%)	0.29 (0.11,0.78)	0.28 (0.10,0.80)
> =1000	1119	66 (5.9%)	2.05 (0.98,4.27)	1.88 (0.83,4.24)	39 (3.5%)	1.69 (0.71,4.03)	1.15 (0.38,3.49)	41 (3.7%)	1.00 (0.45,2.19)	1.03 (0.46,2.34)

*OR* Odds ratio, *aOR* adjusted odds ratio ^a^Adjusted for age, sex, year of data collection, geographic location, governance, CQI participation

## Discussion

To our knowledge, this is the first published report of the quality of social and emotional wellbeing services for families of young Indigenous children in primary care centers. In our study, Indigenous children aged less than 12 months appeared to receive priority for social and emotional wellbeing care but many children did not appear to be receiving services. There was little difference in the documentation of services in remote, rural and urban primary care centers.

In Australia, there are specific national guidelines for social and emotional wellbeing services in primary care centers [3, 5–8], and all our primary care centers used these best practice guidelines for social and emotional wellbeing care. However, social and emotional services did not appear to be well delivered in the primary care centers that participated in our study. Delivery ranged from 11% (advice about nutrition and food security) to 75% (assessment of parent child interaction). Only 55% of families received advice about stimulation of their child and 73% received advice about child behaviour. Almost 25% of families had no clinic follow up or referral for concerns about domestic environment, family support and financial situation, housing condition and food security.

Other Australian studies report even poorer quality of social and emotional wellbeing care [18–22]. Foe example, only 7–40% of women in urban and rural primary care centers in Western Australia received basic advice about family and social supports and maternal depression screening [19, 21]. In contrast, coverage of childhood services such as vaccination has consistently been above 92% at 12 months and 90% at 24 months in Australian Indigenous children [23, 24]. It is likely that families may value the provision of vaccinations more highly than social and emotional wellbeing services. Providers may also find it less challenging to provide a vaccination than to provide a lengthy consultation about social and emotional wellbeing. However, vaccination contacts provide opportunities for services such as anticipatory guidance and social support. The low proportion of families that received social and emotional wellbeing care in our study is disappointing and indicates that there are many missed opportunities for social and emotional wellbeing care in Australian primary care centers.

In our study, we reported better provision of social and emotional wellbeing care in younger (3–11 months) compared to older (12–59 months) children. Other studies have reported better timeliness and coverage in younger children [25, 26]. Low coverage of mental health services and social support for families of school aged children and adolescents has also been reported [27, 28]. However, there appear to be no other published studies that have assessed quality of social and emotional wellbeing services for young children in primary care settings. There are more data on the delivery of primary care services across differing geographic locations. Vaccine delivery, anaemia care and oral health care were reported to be better in small remote communities compared to urban areas [24, 26, 28, 29]. Australian health service providers report that this is due to better communication and engagement with smaller population cohorts [27, 30]. We reported no difference in the documentation of social and emotional wellbeing care across remote, rural and urban communities in our study. However, these analyses were under powered as only four urban and 14 rural clinics were included in our study.

Our study had some limitations. We were only able to include information that was documented in electronic and paper files and we were not able to include observations or interviews with families or service providers. We had small numbers in some subgroups and statistical power was lacking in some analyses. The primary care centers were not randomly selected and participation was voluntary, thus families with fewer social and emotional problems may have attended our clinics. However, our study was designed to measure quality of care rather than disease burden, any selection bias is likely to be non differential, and strengths of our study include our large data set of over 2000 client files, the large number of participating centers and little missing data. We accounted for clustering using multilevel binomial models with an exchangeable correlation structure and robust standard errors were used with primary care center as the clustering variable. Crude and adjusted logistic generalised estimating equations (GEE) were also used to account for loss of independence due to clustering. GEEs do not include measures of ‘goodness-of-fit’ and estimates are sensitive to outliers [15–17]. However, we had no important outliers in our paper and we did not require measures of goodness of fit so we do not consider that these limitations are a problem in our paper. Finally, our definition of quality of care included the explicit delivery of care by a health service provider. This is consistent with the definitions provided by Donabedian in the 1980s and 1990s which are still used by many practitioners today [31, 32].

## Conclusions

In conclusion, our study clearly showed that the families of young Indigenous children receive priority for social and emotional wellbeing care in Australian primary care centers, but many Indigenous children do not receive services. There was little difference in services provided in remote, rural and urban primary care centers.

Our study has implications for policy and program development. In contrast to primary care services in many other countries, Australian Aboriginal community controlled health services have developed from models of comprehensive primary health care and the social and emotional wellbeing of families is at their center [33–35]. However, many Indigenous people have a high burden of physical and mental ill health and Indigenous health services have an important role in proving acute curative care [34]. Indigenous health services are busy places and primary care providers can find it difficult to perform all the preventive services that the clients need. Indeed, only 4% of Indigenous diabetes patients were recently reported to receive screening for depression in an urban Australian health service [36].

Given these constraints, it is encouraging that all the primary care centers in this study included the national best practice guidelines for social and emotional wellbeing care and that important services such as advice about child stimulation, parent child interaction and child behaviour were included. However, implementation and documentation of social and emotional wellbeing care must improve in primary care centers that provide services to Indigenous families. This requires better resourcing, supervision and training to improve the multifaceted skills that are needed to provide these services. It also requires a greater indepth qualitative understanding of the perspectives of Indigenous families and service providers and the barriers to social and emotional wellbeing care for disadvantaged families. Our study also should be replicated in other primary care settings to understand if the delivery of social and emotional wellbeing services differs in populations with low and high levels of vulnerability.

## Additional file

Additional file 1: Table S1. Audit of client files of Australian Indigenous children, health service characteristics, 2012–2014, including denominators; **Table S2.** Audit of client files of Australian Indigenous children, child characteristics, 2012–2014, including denominators; **Table S3.** Audit of client files of Australian Indigenous children, documentation of social and emotional wellbeing services, 2012–2014, including denominators; **Table S4.** Audit of client files of Australian Indigenous children, CQI participation, 2012–2014, including denominators. (DOCX 49 kb)

## Abbreviations

ABCD: Audit and Best Practice for Chronic Disease
ARIA: Accessibility/ Remoteness Index of Australia
CQI: Continuous quality improvement
GEE: Generalised estimating equations
HREC: Human Research Ethics Committee
OR: Odds ratio
