# Supplementary material for: Quality of social and emotional wellbeing services for families of young Indigenous children attending primary care centers; a cross sectional analysis
Source: BMC Health Serv Res. 2018 Feb 9;18:100. doi: 10.1186/s12913-018-2883-6 (PMC5807859; doi:10.1186/s12913-018-2883-6)
Supplement: Supplementary file 1 — Audit of client files of Australian Indigenous children, health service characteristics, 2012–2014, including denominators; Table S2. Audit of client files of Australian Indigenous children, child characteristics, 2012–2014, including denominators; Table S3. Audit of client files of Australian Indigenous children, documentation of social and emotional wellbeing services, 2012–2014, including denominators; Table S4. Audit of client files of Australian Indigenous children, CQI participation, 2012–2014, including denominators. (DOCX 49 kb) [file 12913_2018_2883_MOESM1_ESM.docx]

**ADDITIONAL FILES**

**Table S1 Audit of client files of Australian Indigenous children, health service characteristics, 2012-2014, including denominators**

**Table S2 Audit of client files of Australian Indigenous children, child characteristics, 2012-2014, including denominators**

**Table S3 Audit of client files of Australian Indigenous children, documentation of social and emotional wellbeing services, 2012-2014, including denominators**

**Table S4 Audit of client files of Australian Indigenous children, CQI participation, 2012-2014, including denominators**

**Table S1 Audit of client files of Australian Indigenous children, health service characteristics, 2012-2014, including denominators**

|  | |  |  | **Geographic location of health service** | | | | | | **Age of child** | | | | | |
| --- | --- | --- | --- | --- | --- | --- | --- | --- | --- | --- | --- | --- | --- | --- | --- |
|  | | **Total risk** | | **Remote** | | **Rural** | | **Urban** | | **3-11months** | | **12-23months** | | **24-59months** | |
|  |  | Denominator | n (%) | Denominator | n (%) | Denominator | n (%) | Denominator | n (%) | Denominator | n (%) | Denominator | n (%) | Denominator | n (%) |
| Total audits | | 2466 |  | 2010 |  | 371 |  | 85 |  | 609 |  | 532 |  | 1325 |  |
| CQI participation (number of audits completed) | |  |  |  |  |  |  |  |  |  |  |  |  |  |  |
|  | 1 | 2466 | 410 (16.6%) | 2010 | 256 (12.7%) | 371 | 133 (35.8%) | 85 | 21 (24.7%) | 609 | 99 (16.3%) | 532 | 93 (17.5%) | 1325 | 218 (16.5%) |
|  | 2 | 2466 | 569 (23.1%) | 2010 | 475 (23.6%) | 371 | 84 (22.6%) | 85 | 10 (11.8%) | 609 | 138 (22.7%) | 532 | 120 (22.6%) | 1325 | 311 (23.5%) |
|  | > = 3 | 2466 | 1487 (60.3%) | 2010 | 1279 (63.6%) | 371 | 154 (41.5%) | 85 | 54 (63.5%) | 609 | 372 (61.1%) | 532 | 319 (60.0%) | 1325 | 796 (60.1%) |
| Governance | |  |  |  |  |  |  |  |  |  |  |  |  |  |  |
|  | Aboriginal community controlled health service | 2466 | 573 (23.2%) | 2010 | 319 (15.9%) | 371 | 224 (60.4%) | 85 | 30 (35.3%) | 609 | 133 (21.8%) | 532 | 118 (22.2%) | 1325 | 322 (24.3%) |
|  | Government health service | 2466 | 1893 (76.8%) | 2010 | 1691 (84.1%) | 371 | 147 (39.6%) | 85 | 55 (64.7%) | 609 | 476 (78.2%) | 532 | 414 (77.8%) | 1325 | 1003 (75.7%) |
| Health service provider who first saw the child | |  |  |  |  |  |  |  |  |  |  |  |  |  |  |
|  | Indigenous health worker | 2466 | 338 (13.7%) | 2010 | 220 (10.9%) | 371 | 94 (25.3%) | 85 | 24 (28.2%) | 609 | 69 (11.3%) | 532 | 67 (12.6%) | 1325 | 202 (15.2%) |
|  | Nurse | 2466 | 1723 (69.9%) | 2010 | 1505 (74.9%) | 371 | 176 (47.4%) | 85 | 42 (49.4%) | 609 | 460 (75.5%) | 532 | 381 (71.6%) | 1325 | 882 (66.6%) |
|  | General practitioner | 2466 | 271 (11.0%) | 2010 | 163 (8.1%) | 371 | 90 (24.3%) | 85 | 18 (21.2%) | 609 | 62 (10.2%) | 532 | 60 (11.3%) | 1325 | 149 (11.2%) |
|  | Other | 2466 | 117 (4.7%) | 2010 | 106 (5.3%) | 371 | 10 (2.7%) | 85 | 1 (1.2%) | 609 | 16 (2.6%) | 532 | 20 (3.8%) | 1325 | 81 (6.1%) |
|  | Missing | 2466 | 17 (0.7%) | 2010 | 16 (0.8%) | 371 | 1 (0.3%) | 85 | 0 (0.0%) | 609 | 2 (0.3%) | 532 | 4 (0.8%) | 1325 | 11 (0.8%) |
| Year of data collection | |  |  |  |  |  |  |  |  |  |  |  |  |  |  |
|  | 2012 | 2466 | 488 (19.8%) | 2010 | 319 (15.9%) | 371 | 148 (39.9%) | 85 | 21 (24.7%) | 609 | 127 (20.9%) | 532 | 107 (20.1%) | 1325 | 254 (19.2%) |
|  | 2013 | 2466 | 1334 (54.1%) | 2010 | 1163 (57.9%) | 371 | 171 (46.1%) | 85 | 0 (0.0%) | 609 | 313 (51.4%) | 532 | 276 (51.9%) | 1325 | 745 (56.2%) |
|  | 2014 | 2466 | 644 (26.1%) | 2010 | 528 (26.3%) | 371 | 52 (14.0%) | 85 | 64 (75.3%) | 609 | 169 (27.8%) | 532 | 149 (28.0%) | 1325 | 326 (24.6%) |
| Population size | |  |  |  |  |  |  |  |  |  |  |  |  |  |  |
|  | <=500 | 2466 | 848 (34.4%) | 2010 | 831 (41.3%) | 371 | 17 (4.6%) | 85 | 0 (0.0%) | 609 | 137 (22.5%) | 532 | 196 (36.8%) | 1325 | 515 (38.9%) |
|  | 501-999 | 2466 | 499 (20.2%) | 2010 | 448 (22.3%) | 371 | 41 (11.1%) | 85 | 10 (11.8%) | 609 | 114 (18.7%) | 532 | 101 (19.0%) | 1325 | 284 (21.4%) |
|  | >=1000 | 2466 | 1119 (45.4%) | 2010 | 731 (36.4%) | 371 | 313 (84.4%) | 85 | 75 (88.2%) | 609 | 358 (58.8%) | 532 | 235 (44.2%) | 1325 | 526 (39.7%) |

**Table S2 Audit of client files of Australian Indigenous children, child characteristics, 2012-2014, including denominators**

|  | |  |  | **Geographic location of health service** | | | | | | **Age of child** | | | | | |
| --- | --- | --- | --- | --- | --- | --- | --- | --- | --- | --- | --- | --- | --- | --- | --- |
|  | | **Total risk** | | **Remote** | | **Rural** | | **Urban** | | **3-11months** | | **12-23months** | | **24-59months** | |
|  |  | Denominator | n (%) | Denominator | n (%) | Denominator | n (%) | Denominator | n (%) | Denominator | n (%) | Denominator | n (%) | Denominator | n (%) |
| Total audits | |  | 2466 |  | 2010 |  | 371 |  | 85 |  | 609 |  | 532 |  | 1325 |
| Sex of child | |  |  |  |  |  |  |  |  |  |  |  |  |  |  |
|  | Male | 2466 | 1249 (50.6%) | 2010 | 1017 (50.6%) | 371 | 189 (50.9%) | 85 | 43 (50.6%) | 609 | 310 (50.9%) | 532 | 272 (51.1%) | 1325 | 667 (50.3%) |
|  | Female | 2466 | 1217 (49.4%) | 2010 | 993 (49.4%) | 371 | 182 (49.1%) | 85 | 42 (49.4%) | 609 | 299 (49.1%) | 532 | 260 (48.9%) | 1325 | 658 (49.7%) |
| Type of child health check completed in the last 12 months | | |  |  |  |  |  |  |  |  |  |  |  |  |  |
|  | Medical benefits schedule (MBS) 715 | 2466 | 999 (40.5%) | 2010 | 847 (42.1%) | 371 | 122 (32.9%) | 85 | 30 (35.3%) | 609 | 246 (40.4%) | 532 | 229 (43.0%) | 1325 | 524 (39.5%) |
|  | Other child health check | 2466 | 648 (26.3%) | 2010 | 507 (25.2%) | 371 | 124 (33.4%) | 85 | 17 (20.0%) | 609 | 181 (29.7%) | 532 | 147 (27.6%) | 1325 | 320 (24.2%) |
|  | Not known / not recorded | 2466 | 819 (33.2%) | 2010 | 656 (32.6%) | 371 | 125 (33.7%) | 85 | 38 (44.7%) | 609 | 182 (29.9%) | 532 | 156 (29.3%) | 1325 | 481 (36.3%) |
| Reason for last clinic attendance | | | |  |  |  |  |  |  |  |  |  |  |  |  |
|  | Acute care | 2466 | 1200 (48.7%) | 2010 | 990 (49.3%) | 371 | 171 (46.1%) | 85 | 39 (45.9%) | 609 | 265 (43.5%) | 532 | 271 (50.9%) | 1325 | 664 (50.1%) |
|  | Vaccination | 2466 | 366 (14.8%) | 2010 | 268 (13.3%) | 371 | 78 (21.0%) | 85 | 20 (23.5%) | 609 | 122 (20.0%) | 532 | 87 (16.4%) | 1325 | 157 (11.8%) |
|  | Child health check | 2466 | 577 (23.4%) | 2010 | 467 (23.2%) | 371 | 92 (24.8%) | 85 | 18 (21.2%) | 609 | 155 (25.5%) | 532 | 112 (21.1%) | 1325 | 310 (23.4%) |
|  | Other | 2466 | 323 (13.1%) | 2010 | 285 (14.2%) | 371 | 30 (8.1%) | 85 | 8 (9.4%) | 609 | 67 (11.0%) | 532 | 62 (11.7%) | 1325 | 194 (14.6%) |

**Table S3 Audit of client files of Australian Indigenous children, documentation of social and emotional wellbeing services, 2012-2014, including denominators**

|  |  |  |  | **Geographic location of health service** | | | | | | **Age of child** | | | | | |
| --- | --- | --- | --- | --- | --- | --- | --- | --- | --- | --- | --- | --- | --- | --- | --- |
|  |  | **Total risk** | | **Remote** | | **Rural** | | **Urban** | | **3-11 months** | | **12-23 months** | | **24-59 months** | |
|  |  | Denominator | n (%) | Denominator | n (%) | Denominator | n (%) | Denominator | n (%) | Denominator | n (%) | Denominator | n (%) | Denominator | n (%) |
| Assessment | |  |  |  |  |  |  |  |  |  |  |  |  |  |  |
|  | Assessment of parent-child interaction | 1628 | 1216 (74.7%) | 1249 | 986 (78.9%) | 303 | 198 (65.4%) | 76 | 32 (42.1%) | 608 | 516 (84.9%) | 531 | 389 (73.3%) | 489 | 311 (63.6%) |
| Anticipatory guidance | |  |  |  |  |  |  |  |  |  |  |  |  |  |  |
|  | Advice about domestic/social environment | 2466 | 1544 (62.6%) | 2010 | 1276 (63.5%) | 371 | 221 (59.6%) | 85 | 47 (55.3%) | 609 | 427 (70.1%) | 532 | 368 (69.2%) | 1325 | 749 (56.5%) |
|  | Advice about social/family support | 2333 | 1410 (60.4%) | 1912 | 1162 (60.8%) | 340 | 205 (60.3%) | 81 | 43 (53.1%) | 609 | 414 (68.0%) | 532 | 367 (69.0%) | 1192 | 629 (52.8%) |
|  | Advice about financial situation | 1373 | 236 (17.2%) | 1254 | 210 (16.8%) | 89 | 5 (5.6%) | 30 | 21 (70.0%) | 336 | 79 (23.5%) | 285 | 62 (21.8%) | 752 | 95 (12.6%) |
|  | Advice about housing condition | 2466 | 1140 (46.2%) | 2010 | 939 (46.7%) | 371 | 169 (45.6%) | 85 | 32 (37.7%) | 609 | 327 (53.7%) | 532 | 255 (47.9%) | 1325 | 558 (42.1%) |
|  | Advice about food security | 963 | 102 (10.6%) | 752 | 73 (9.7%) | 187 | 23 (12.3%) | 24 | 6 (25.0%) | 246 | 39 (15.9%) | 209 | 29 (13.9%) | 508 | 34 (6.7%) |
|  | Advice about physical and mental stimulation of child | 2322 | 1279 (55.1%) | 2006 | 1137 (56.7%) | 262 | 102 (38.9%) | 54 | 40 (74.1%) | 570 | 366 (64.2%) | 503 | 312 (52.0%) | 1249 | 601 (48.1%) |
|  | Advice about child behaviour (e.g temper tantrums, sleep disturbance) | 963 | 703 (73.0%) | 752 | 584 (77.7%) | 187 | 102 (54.6%) | 24 | 17 (70.8%) | 246 | 211 (85.8%) | 209 | 170 (81.3%) | 508 | 322 (63.4%) |
| Follow up of problems and concerns | |  |  |  |  |  |  |  |  |  |  |  |  |  |  |
|  | Clinic follow up of problems with domestic environment | 111 | 71 (64.0%) | 86 | 54 (62.8%) | 18 | 13 (72.2%) | 7 | 4 (57.1%) | 25 | 16 (64.0%) | 30 | 22 (73.3%) | 56 | 33 (58.9%) |
|  | Referral for problems with domestic environment | 111 | 71 (64.0%) | 86 | 54 (62.8%) | 18 | 14 (77.8%) | 7 | 3 (42.9%) | 25 | 14 (56.0%) | 30 | 19 (63.3%) | 56 | 38 (67.9%) |
|  | No clinic follow up or referral for problems with domestic environment | 111 | 29 (26.1%) | 86 | 23 (26.7%) | 18 | 3 (16.7%) | 7 | 3 (42.9%) | 25 | 8 (32.0%) | 30 | 7 (23.3%) | 56 | 14 (25.0%) |
|  | Clinic follow up for family and financial support | 63 | 39 (61.9%) | 45 | 29 64.4%) | 12 | 7 (58.3%) | 6 | 3 (50.0%) | 13 | 8 (61.5%) | 21 | 14 (66.7%) | 29 | 17 (58.6%) |
|  | Referral for family and financial support | 63 | 39 (61.9%) | 45 | 27 (60.0%) | 12 | 9 (75.0%) | 6 | 3 (50.0%) | 13 | 8 (61.5%) | 21 | 11 (52.4%) | 29 | 20 (69.0%) |
|  | No clinic follow up or referral for family and financial support | 63 | 17 (27.0%) | 45 | 13 (28.9%) | 12 | 2 (16.7%) | 6 | 2 (33.3%) | 13 | 4 (30.8%) | 21 | 7 (33.3%) | 29 | 6 (20.7%) |
|  | Clinic follow up for housing condition or food security | 80 | 33 (41.3%) | 69 | 26 (37.7%) | 8 | 6 (75.0%) | 3 | 1 (33.3%) | 18 | 7 (38.9%) | 22 | 9 (40.9%) | 40 | 17 (42.5%) |
|  | Referral for housing condition or food security | 80 | 49 (61.3%) | 69 | 43 (62.3%) | 8 | 4 (50.0%) | 3 | 2 (66.7%) | 18 | 10 (55.6%) | 22 | 14 (63.6%) | 40 | 25 (62.5%) |
|  | No clinic follow up or referral for housing condition or food security | 80 | 25 (31.3%) | 69 | 22 (31.9%) | 8 | 2 (25.0%) | 3 | 1 (33.3%) | 18 | 6 (33.3%) | 22 | 8 (36.4%) | 40 | 11 (27.5%) |
| Composite measure of quality of care (families received advice about domestic environment, social support, housing condition  and child stimulation) | | 2189 | 712 (32.5%) | 1908 | 622 (32.6%) | 231 | 68 (29.4%) | 50 | 22 (44.0%) | 570 | 225 (39.5%) | 503 | 174 (34.6%) | 1116 | 313 (28.0%) |

*Families received advice about domestic environment, social support, housing condition and child stimulation

**Table S4 Audit of client files of Australian Indigenous children, CQI participation, 2012-2014, including denominators**

|  | | |  |  | **CQI participation (number of annual audit rounds**  **implemented by health service)** | | | | | | |
| --- | --- | --- | --- | --- | --- | --- | --- | --- | --- | --- | --- |
|  | | | **Total** | | **1** | | **2** | | **> 3** | | |
|  |  |  | Denominator | n (%) | Denominator | n (%) | Denominator | n (%) | Denominator | n (%) |  |
| **Total audits** | | | 2466 | 2466 | 410 | 410 | 569 | 569 | 1487 | 1487 |  |
| **Child characteristics** | | |  |  |  |  |  |  |  |  |  |
| Sex of child | | |  |  |  |  |  |  |  |  |  |
|  | | Male | 2466 | 1249 (50.6%) | 410 | 202 (49.3%) | 569 | 283 (49.7%) | 1487 | 764 (51.4%) |  |
|  | | Female | 2466 | 1217 (49.4%) | 410 | 208 (50.7%) | 569 | 286 (50.3%) | 1487 | 723 (48.6%) |  |
| Type of child health check completed in the last 12 months | | |  |  |  |  |  |  |  |  |  |
|  | | Medical benefits schedule (MBS) 715 | 2466 | 999 (40.5%) | 410 | 115 (28.0%) | 569 | 212 (37.3%) | 1487 | 672 (45.2%) |  |
|  | | Other child health check | 2466 | 648 (26.3%) | 410 | 144 (35.1%) | 569 | 88 (15.5%) | 1487 | 416 (28.0%) |  |
|  | | Not known / not recorded | 2466 | 819 (33.2%) | 410 | 151 (36.8%) | 569 | 269 (47.3%) | 1487 | 399 (26.8%) |  |
| Reason for last clinic attendance | | |  |  |  |  |  |  |  |  |  |
|  | | Acute care | 2466 | 1200 (48.7%) | 410 | 161 (39.3%) | 569 | 284 (49.9%) | 1487 | 755 (50.8%) |  |
|  | | Vaccination | 2466 | 366 (14.8%) | 410 | 64 (15.6%) | 569 | 89 (15.6%) | 1487 | 213 (14.3%) |  |
|  | | Child health check | 2466 | 577 (23.4%) | 410 | 105 (25.6%) | 569 | 117 (20.6%) | 1487 | 355 (23.9%) |  |
|  | | Other | 2466 | 323 (13.1%) | 410 | 80 (19.5%) | 569 | 79 (13.9%) | 1487 | 164 (11.0%) |  |
| **Health service characteristics** | | |  |  |  |  |  |  |  |  |  |
| Governance | | |  |  |  |  |  |  |  |  |  |
|  | | Aboriginal community controlled health service | 2466 | 573 (23.2%) | 410 | 77 (18.8%) | 569 | 182 (32.0%) | 1487 | 314 (21.1%) |  |
|  | | Government health service | 2466 | 1893 (76.8%) | 410 | 333 (81.2%) | 569 | 387 (68.0%) | 1487 | 1173 (78.9%) |  |
| Health service provider who first saw the child | | |  |  |  |  |  |  |  |  |  |
|  | | Indigenous health worker | 2466 | 338 (13.7%) | 410 | 21 (5.1%) | 569 | 88 (15.5%) | 1487 | 229 (15.4%) |  |
|  | | Nurse | 2466 | 1723 (69.9%) | 410 | 286 (69.8%) | 569 | 430 (75.6%) | 1487 | 1007 (67.7%) |  |
|  | | General practitioner | 2466 | 271 (11.0%) | 410 | 81 (19.8%) | 569 | 27 (4.7%) | 1487 | 163 (11.0%) |  |
|  | | Other | 2466 | 117 (4.7%) | 410 | 21 (5.1%) | 569 | 24 (4.2%) | 1487 | 72 (4.8%) |  |
|  | | Not stated | 2466 | 17 (0.7%) | 410 | 1 (0.2%) | 569 | 0 (0.0%) | 1487 | 16 (1.1%) |  |
| Year of data collection | | |  |  |  |  |  |  |  |  |  |
|  | | 2012 | 2466 | 488 (19.8%) | 410 | 196 (47.8%) | 569 | 84 (14.8%) | 1487 | 208 (14.0%) |  |
|  | | 2013 | 2466 | 1334 (54.1%) | 410 | 155 (37.8%) | 569 | 274 (48.2%) | 1487 | 905 (60.9%) |  |
|  | | 2014 | 2466 | 644 (26.1%) | 410 | 59 (14.4%) | 569 | 211 (37.1%) | 1487 | 374 (25.2%) |  |
| Population size | | |  |  |  |  |  |  |  |  |  |
|  | <=500 | | 2466 | 848 (34.4%) | 410 | 101 (24.6%) | 569 | 175 (30.8%) | 1487 | 572 (38.5%) |  |
|  | 501-999 | | 2466 | 499 (20.2%) | 410 | 104 (25.4%) | 569 | 92 (16.2%) | 1487 | 303 (20.4%) |  |
|  | >=1000 | | 2466 | 1119 (45.4%) | 410 | 205 (50.0%) | 569 | 302 (53.1%) | 1487 | 612 (41.2%) |  |
| **Social and emotional wellbeing care** | | |  |  |  |  |  |  |  |  |  |
| Assessment | | |  |  |  |  |  |  |  |  |  |
|  | | Assessment of parent-child interaction | 1628 | 1216 (74.7%) | 260 | 155 (59.6%) | 315 | 231 (73.3%) | 1053 | 830 (78.8%) |  |
| Anticipatory guidance | | |  |  |  |  |  |  |  |  |  |
|  | | Advice about domestic/social environment | 2466 | 1544 (62.6%) | 410 | 201 (49.0%) | 569 | 293 (51.5%) | 1487 | 1050 (70.6%) |  |
|  | | Advice about social/family support | 2333 | 1410 (60.4%) | 380 | 182 (47.9%) | 567 | 281 (49.6%) | 1386 | 947 (68.3%) |  |
|  | | Advice about financial situation | 1373 | 236 (17.2%) | 256 | 61 (23.8%) | 455 | 35 (7.7%) | 662 | 140 (21.1%) |  |
|  | | Advice about housing condition | 2466 | 1140 (46.2%) | 410 | 167 (40.7%) | 569 | 245 (43.1%) | 1487 | 728 (49.0%) |  |
|  | | Advice about food security | 963 | 102 (10.6%) | 116 | 10 (8.6%) | 20 | 0 (0.0%) | 827 | 92 (11.1%) |  |
|  | | Advice about physical and mental stimulation of child | 2322 | 1279 (55.1%) | 372 | 135 (36.3%) | 475 | 222 (46.7%) | 1475 | 922 (62.5%) |  |
|  | | Advice about child behaviour (e.g temper tantrums, sleep disturbance) | 963 | 703 (73.0%) | 116 | 65 (56.0%) | 20 | 20 (100.0%) | 827 | 618 (74.7%) |  |
| Follow up of problems and concerns | | |  |  |  |  |  |  |  |  |  |
|  | | Clinic follow up of problems with domestic environment | 111 | 71 (64.0%) | 19 | 14 (73.7%) | 17 | 7 (41.2%) | 75 | 50 (66.7%) |  |
|  | | Referral for problems with domestic environment | 111 | 71 (64.0%) | 19 | 14 (73.7%) | 17 | 7 (41.2%) | 75 | 50 (66.7%) |  |
|  | | No clinic follow up or referral for problems with domestic environment | 111 | 29 (26.1%) | 19 | 3 (15.8%) | 17 | 8 (47.1%) | 75 | 18 (24.0%) |  |
|  | | Clinic follow up for family and financial support | 63 | 39 (61.9%) | 22 | 12 (54.5%) | 7 | 4 (57.1%) | 34 | 23 (67.6%) |  |
|  | | Referral for family and financial support | 63 | 39 (61.9%) | 22 | 15 (68.2%) | 7 | 3 (42.9%) | 34 | 21 (61.8%) |  |
|  | | No clinic follow up or referral for family and financial support | 63 | 17 (27.0%) | 22 | 7 (31.8%) | 7 | 2 (28.6%) | 34 | 8 (23.5%) |  |
|  | | Clinic follow up for housing condition or food security | 80 | 33 (41.3%) | 23 | 11 (47.8%) | 12 | 2 (16.7%) | 45 | 20 (44.4%) |  |
|  | | Referral for housing condition or food security | 80 | 49 (61.3%) | 23 | 12 (52.2%) | 12 | 5 (41.7%) | 45 | 32 (71.1%) |  |
|  | | No clinic follow up or referral for housing condition or food security | 80 | 25 (31.3%) | 23 | 9 (39.1%) | 12 | 6 (50.0%) | 45 | 10 (22.2%) |  |
| Composite measure of quality of care* | | | 2189 | 712 (32.5%) | 342 | 81 (23.7%) | 473 | 116 (24.5%) | 1374 | 515 (37.5%) |  |

* Families received advice about domestic environment, social support, housing condition and child stimulation
